# Rainy Day Reaction: Human West Nile Viruses Cases Respond to Weather Patterns

**DOI:** 10.1289/ehp.117-a311b

**Published:** 2009-07

**Authors:** Bob Weinhold

**Affiliations:** **Bob Weinhold**, MA, has covered environmental health issues for numerous outlets since 1996. He is a member of the Society of Environmental Journalists

Piecemeal evidence suggests weather may have played a role in the rapid spread of West Nile virus (WNV) across the United States and into Canada and Central America following its detection in New York City in 1999. A team of U.S. and Canadian researchers has looked more comprehensively at the evidence by analyzing a spectrum of weather factors for 17 climatically diverse states, and found several significant links with the incidence of human WNV cases **[*EHP*** **117:1049–1052; Soverow et al.]**. The analysis was based on 16,298 WNV cases reported to the Centers for Disease Control and Prevention from 2001 to 2005, as well as year-round temperature, precipitation, and dew point data from 351 weather stations in close proximity to the infected people.

A 12°F increase in maximum daily temperature was associated with a 45–72% increase in WNV case reports within a 1-month period. Precipitation was also associated with WNV, which increased 29–66% in association with a single-day rainfall of at least 50 mm within 3 weeks of diagnosis. Smaller amounts of precipitation were associated with smaller increases in WNV cases, consistent with a dose–response effect. Increases in cumulative weekly precipitation and mean weekly dew point temperature (a measure of relative humidity) were also associated with an increase in WNV cases.

The findings, which hold up across season and location, generally mesh with what is known about the biology of WNV, humans, mosquito vectors, and bird reservoir hosts. The authors write that additional research will be needed to address some limitations of their work—notably gaps in data from a number of geographic regions and the influence of localized interactions of factors such as bird populations, vegetation, mosquito control efforts, and acquired immunity in both humans and animals. If these weather–disease links are confirmed, and if climate changes in North America unfold as predicted with increases in temperature and precipitation, public health officials may be better able to prevent or mitigate outbreaks in the future.

